# BrainWideBench: Benchmarking large-scale pretraining and across-animal transfer in multi-region neural recordings

**Published:** 2026-09-18

**Authors:** Alexandre Andre, Shivashriganesh P. Mahato, Vinam Arora, Keshav Balaji, Divyansha Lachi, Nanda H. Krishna, Jingyun Xiao, Yizi Zhang, Ximeng Mao, Wenrui Ma, Han Yu, International Brain Laboratory, Daniel Birman, Niccolò Bonacchi, Gaelle A. Chapuis, Joana A. Catarino, Felicia Davatolhagh, Mayo Faulkner, Laura Freitas-Silva, Fei Hu, Julia M. Huntenburg, Anup Khanal, Inês Laranjeira, Petrina Lau, Guido T. Meijer, Nathaniel J. Miska, Jean-Paul Noel, Alejandro Pan-Vazquez, Georg Raiser, Cyrille Rossant, Karolina Z. Socha, Anne E. Urai, Miles J. Wells, Steven J. West, Olivier Winter, Blake Richards, Guillaume Lajoie, Cole Hurwitz, Mehdi Azabou, Matthew R. Whiteway, Liam Paninski, Eva L. Dyer

**Affiliations:** 1 University of Pennsylvania,; 2 Mila,; 3 Université de Montréal,; 4 Stanford University,; 5 Columbia University,; 6 Allen Institute,; 7 William James Center for Research,; 8 ISPA - Instituto Universitário,; 9 University of Geneva,; 10 Karolinska Institutet,; 11 UCLA,; 12 University College London,; 13 Champalimaud Foundation,; 14 Lingang Laboratory,; 15 The Chinese University of Hong Kong,; 16 Donders Institute,; 17 University of Minnesota,; 18 Princeton University,; 19 Leiden University,; 20 McGill University,; 21 IBM

## Abstract

Advances in large-scale neural recording have made it possible to collect data across many animals and distributed brain regions, raising the question of whether this scale can be exploited to learn general-purpose neural representations transferable across diverse downstream tasks. Yet, progress toward this goal has been limited by fragmented evaluation protocols and a narrow focus on individual task domains. Here, we present BrainWideBench, a benchmark for evaluating across-animal transfer on multi-region neural recordings, built on the International Brain Laboratory Brainwide Map dataset of neural and behavioral recordings spanning 276 brain regions from 139 mice performing a sensory-guided decision-making task. The benchmark is organized around three complementary task suites that evaluate whether learned representations support downstream decoding of behavior, can predict masked or future neural activity, and can recover biologically meaningful anatomical organization. With this benchmark, we systematically evaluate pretraining methods across transfer settings, including finetuning on downstream objectives and zero-shot generalization to unseen animals. Our results confirm pretraining improves performance over matched single-session baselines, but we show current methods exhibit heterogeneity in transfer capabilities: gains depend strongly on the alignment between pretraining objectives and downstream tasks. No single approach performs uniformly well across all three suites, and most methods are designed to only address a subset of them. Together, these findings suggest that learning representations that jointly generalize across behavior, dynamics, and anatomy remains an open challenge. By providing a unified and reproducible evaluation suite, BrainWideBench establishes a framework for measuring progress toward general-purpose models of the mouse brain. Code is available at this link.

## 1 Introduction

Over the past decade, advances in large-scale neural recording technologies have fundamentally reshaped systems neuroscience. High-density electrophysiology [1–3] now enables simultaneous recording of hundreds to thousands of individual neurons across multiple brain regions, producing brain-wide datasets up to single-cell, single-spike resolution across many animals performing complex behaviors [4–9]. For the first time, it is possible to observe distributed neural computation across the brain at scale. These datasets promise insight into how perception, decision-making, memory, and action emerge from coordinated population dynamics spanning cortex, thalamus, hippocampus, and beyond. Yet the field remains constrained by analysis approaches that operate at the level of individual sessions or isolated regions, limiting our ability to study shared principles from the growing body of multi-animal recordings [10].

Foundation models offer a fundamentally different way to approach these datasets [11, 12]. Rather than training separate models for each animal, region, or task, foundation models seek to learn shared representations across heterogeneous recordings by extracting patterns that recur across individuals and brain areas [13–20]. In this view, each recording is not an isolated experiment but a partial observation of a broader underlying computational system. Scaling across animals and anatomical coverage may allow models to distill invariant features of neural dynamics that are not visible within a single session or region. Similar scaling efforts in language and vision have revealed predictable trends in performance and emergent capabilities as data and model size increase [21, 22]. Whether analogous principles hold for neural population activity, and whether large-scale training can uncover shared computational motifs across the brain, remains an open question.

Answering this question requires more than methodological advancements in large-scale modeling of neural data; it requires benchmarks that make scaling and progress measurable. In areas ranging from language modeling, mathematics, and coding to scientific machine learning domains such as biology or materials science, standardized evaluation suites have driven rapid progress by providing curated datasets, unified metrics, and reproducible splits that enable systematic comparison across methods [23–30]. These benchmarks have made it possible to quantify scaling behavior, measure transfer, and detect emergent capabilities.

Existing benchmarks for spike-resolution neural recordings either focus on a small number of animals with limited anatomical coverage or a single predictive task (e.g., neural encoding) [31–33], limiting the ability to study generalization across animals or tasks and distributed computation at scale. To our knowledge, no current benchmark exists for *testing across-subject transfer in multi-region neural recordings*, where the goal is to first build a model on pretrained representations and deploy it across *multiple downstream task families* within a unified framework. As a result, the field lacks a standardized platform for measuring how well pretrained models transfer and whether improvements reflect genuine representation learning or task-specific optimization.

To address this gap, we introduce BrainWideBench, a benchmark spanning multiple downstream tasks, built on the International Brain Laboratory’s Brainwide Map dataset [8]. The dataset comprises over 600 hours of neuron-level, single-spike recordings spanning 276 anatomical regions, from 139 mice performing a vision-guided decision-making task with simultaneous behavioral measurements. BrainWideBench defines three complementary families of tasks that probe distinct properties. The first task suite asks whether *behaviorally-relevant variables can be decoded* from neural activity. The second task suite asks whether *neural activity can be predicted* from other neurons or past activity. The third task suite asks whether *brain regions can be directly inferred* from neural activity. We establish a standardized split for pretraining on many animals and transferring to a held-out set of animals on downstream tasks spanning the three families. To characterize the current landscape of neural population models, we evaluate a diverse set of baselines spanning transformer-based and state-space-based architectures. This evaluation framework moves beyond evaluation on a single task at a time, toward a more comprehensive notion of representation quality that reflects generalization across animals, brain regions, and tasks.

By combining standardized splits, evaluation of animal-level transfer, and brain-wide anatomical coverage, BrainWideBench establishes a platform for studying scaling laws, transfer properties, and curriculum learning for pretrained models. Along with the task suites, we provide comprehensive session, probe, and unit-level quality control (QC) metadata. Baselines are trained under common QC standards, and full metadata is provided to enable research into how data quality shapes pretraining and model robustness. We envision this benchmark as a step toward generalizable, brain-wide models that capture shared principles of neural computation across individuals.

**The main contributions of this work include:**

- A new benchmark for evaluating how models pretrained on neural recordings **transfer across diverse downstream tasks**, organized around three complementary task suites spanning behavior and stimulus decoding, neural activity prediction, and anatomical identification. Together, these tasks evaluate whether learned representations capture transferable behavioral, dynamical, and anatomical structure across animals and recording sessions.
- **A curated dataset derived from the IBL Brainwide Map**, accompanied by standardized train/test splits and quality-control metadata at the session, probe, and unit level. This supports reproducible evaluation while enabling systematic study of how scale, recording variability, and data quality impact pretraining and transfer.
- **A comprehensive empirical study** of supervised, and self-supervised models, spanning both single-session baselines and recent large-scale pretraining approaches. Through evaluations across behavioral, neural, and anatomical tasks, we identify key strengths and limitations of current approaches.

## 2 Motivation

### 2.1 What are the goals of this benchmark?

Large-scale modeling in neuroscience is rapidly expanding, with a growing number of works exploring pretraining and transfer for neural data [13, 14, 16, 17, 33]. These approaches have demonstrated promising gains across decoding, forecasting, and cross-session transfer, suggesting that neural activity contains substantial shared structure that can be leveraged through large-scale learning. However, evaluation remains highly fragmented: individual works often introduce new datasets, train/test splits, modalities, or downstream tasks, making it difficult to determine whether improvements reflect genuinely more general representations or simply differences in evaluation protocols.

At the same time, an important open question remains: *what would a truly generalist neural foundation model look like?* Such a model should do more than solve a single decoding or forecasting problem. Instead, it should learn representations that support a diverse set of downstream objectives, including behavioral decoding, neural activity prediction, and anatomical identification, while generalizing across animals, recording sessions, and experimental conditions.

The goal of BrainWideBench is therefore two-fold: to provide a standardized and reproducible testbed for developing and comparing pretrained neural models, and to define a set of core downstream tasks that reflect fundamental properties of neural representations. To this end, BrainWideBench is organized into three complementary task suites. The first evaluates *behavioral decoding*, testing whether representations encode task-relevant variables that generalize across animals and sessions. The second measures *neural activity prediction*, assessing whether models capture structured spatiotemporal population dynamics. The third evaluates *neural identity prediction*, probing whether anatomical organization (brain region) emerges in pretrained representations and transfers without supervision. Together, these task suites test whether a single set of representations, pretrained across many mice and brain regions, transfers to held-out mice on multiple downstream tasks and under a standardized experimental setting.

### 2.2 Why this dataset?

The International Brain Laboratory (IBL) Brainwide Map [8] provides a rich multi-region dataset that is ideal for benchmarking pretraining strategies for multi-animal, large-scale neural recordings. It is a large-scale, multi-laboratory neurophysiology resource that captures brain-wide neural activity along with detailed behavioral measurements during a standardized decision-making task (Figure 2) [34]. A key advantage of this dataset is its scale and consistency. Data were collected across a coordinated collaboration of laboratories using shared experimental protocols, hardware, and preprocessing pipelines, enabling reproducible comparisons across animals, sessions, and brain regions [34–37]. This level of standardization is critical for evaluating generalization, as it reduces confounds introduced by dataset-specific variability.

The behavioral task involves visual decision-making, where mice respond to Gabor stimuli presented at varying contrasts and spatial locations. Structured manipulations of stimulus visibility and priors on spatial placement induce variability in perception, decision-making, and belief states, providing a rich substrate for probing behaviorally relevant representations. Neural activity is recorded using Neuropixels probes [1], yielding high-density spiking data from hundreds of neurons simultaneously. Across the dataset, recordings span 688 probe insertions in 139 mice, covering 276 anatomical regions and yielding over 600,000 units if no filters are applied (see Table A1 for unit counts under various filtering criteria). This brain-wide coverage enables evaluation of representations across diverse functional circuits, including sensory, motor, and cognitive systems.

In addition to neural recordings, the dataset includes synchronized behavioral signals such as trial structure, stimulus parameters, wheel movements, and video-based pose estimation [38]. Standardized preprocessing pipelines, including spike sorting [39] and video analysis [40], align these modalities into a unified format. The dataset is accessible through the Open Neurophysiology Environment (ONE) API [36], which provides a consistent interface for querying data across sessions, probes, and regions^2^. This enables scalable and reproducible benchmarking workflows. Together, the scale, standardization, multimodality, and brain-wide coverage of the IBL dataset make it particularly well suited for our benchmark.

## 3 BrainWideBench Task Suites

In the following sections, we detail the task suites that constitute the BrainWideBench, outlining motivations and specific design choices around them, as well as representative baselines. We characterize pretrained baselines in terms of their training objective: *task-suite-supervised* (TSS) models pretrain directly on the downstream tasks, and *task-suite-unsupervised* (TSU) models utilize a pretraining objective disjoint from the downstream tasks. To reduce complexity and standardize evaluation, models are tested on a single-session, single-task basis for each task suite. At the same time, users are free to explore different fine-tuning strategies, few-shot adaptation, or fully zero-shot transfer during evaluation. This design supports systematic study of scaling behavior, across-animal transfer, and representation learning in large-scale pretraining for neural data.

### 3.1 Across-animal transfer as a test of generalization

**Figure 1:**
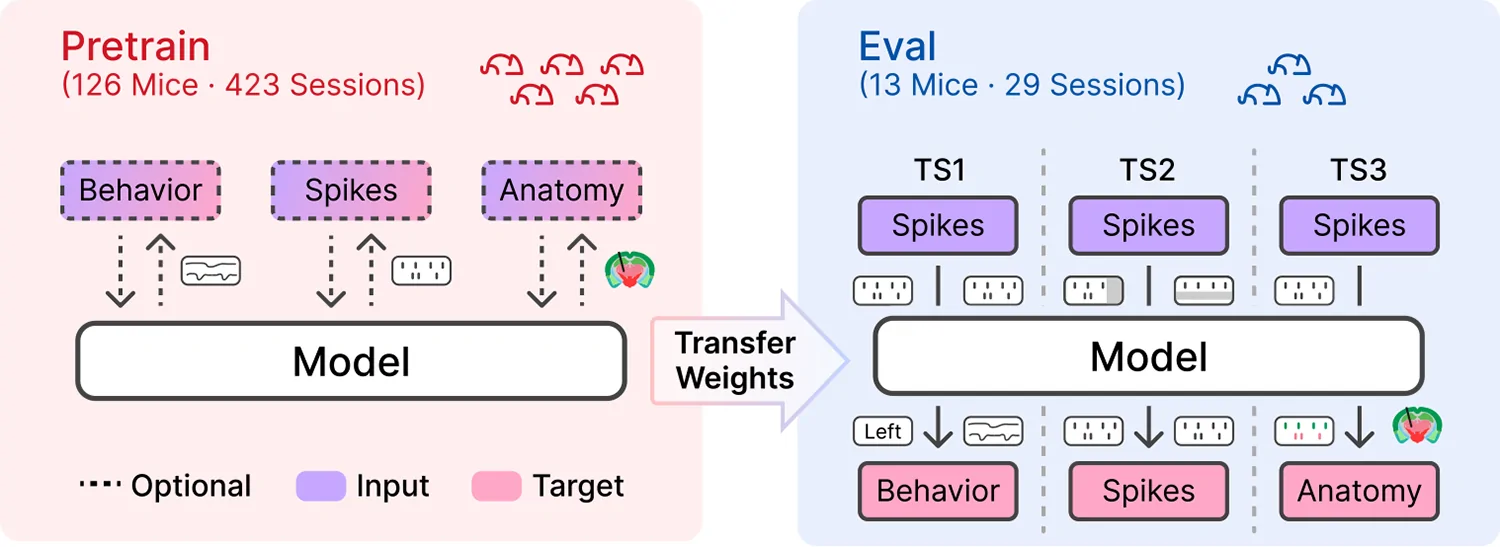
Overview of the benchmark. (Left) During pretraining, models have access to a large pretraining set with 126 mice that includes behavior, spike sorted neural recordings, and anatomical labels (e.g., brain region), to learn generalized representations. Models are pretrained and evaluated (Right) on a held-out set of 13 animals across three task suites: TS1 (decoding), TS2 (neural activity prediction), and TS3 (brain region classification).

**Figure 2:**
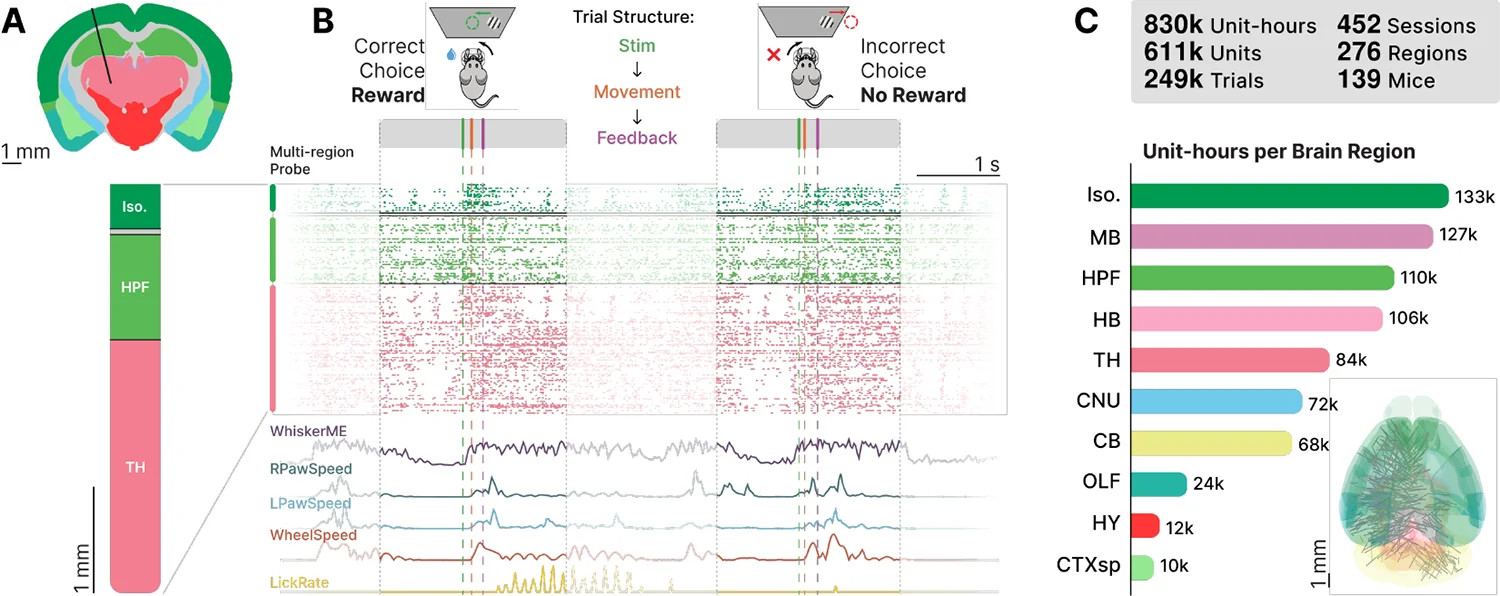
Overview of IBL Brainwide Map Dataset. (A) Multi-region probes are inserted into the brain and mapped onto a common coordinate framework (CCF) atlas to map individual neural spike sorted units to different anatomical regions. (B) An example of neural and behavioral data captured simultaneously while the mouse tries to control a wheel to move a visual stimulus from either the left or right side of the screen to the center. We capture paws and wheel speed, whisker motion, and licking from the water spout. After a correct trial, the mouse is rewarded with water. (C) Data from individual probes and sessions are aggregated which in total amounts to over 800,000 neuron hours.

A central challenge in modeling neural data is learning representations that transfer across animals. Each recording provides only a partial and noisy view of a shared latent system underlying behavior and neural computation [41, 42]. Given the broad anatomical coverage of the Brainwide Map [8], successful transfer further requires models to capture neural structure and dynamics distributed across the brain. To evaluate this capability, BrainWideBench uses a strict animal-level split between pretraining and evaluation datasets (Appendix C), illustrated schematically in Figure 1.

This split structure enables evaluation of three complementary capabilities: (1) large-scale representation learning during pretraining, (2) adaptation to new animals with limited supervision, and (3) zero-shot generalization without task-specific fine-tuning. The pretraining corpus consists of recordings from 126 animals spanning 423 sessions and 274 brain regions, with access to all available supervision, including behavioral labels and anatomical annotations. Evaluation is performed on disjoint recordings from 13 held-out animals spanning 29 sessions and 124 regions, ensuring that models must generalize to unseen individuals rather than relying on subject-specific structure.

### 3.2 Task Suite 1: Behavior Prediction

#### TS1: Motivation.

A central goal in systems neuroscience is to understand how neural activity gives rise to behavior [43, 44]. Behavior decoding provides a way to probe this relationship by evaluating how well models can extract behaviorally relevant information from neural population activity. From a neuroscience perspective, successful decoding enables the identification of behaviorally relevant signals distributed across populations, offering insights into how information is represented and transformed across circuits [8, 34]. From an applications perspective, behavior decoding underpins brain-computer interfaces, neural prosthetics, and assistive technologies, where accurate inference of intent or state from neural activity is critical for real-time interaction [32, 45, 46]. In the context of large-scale pretraining, decoding tasks test whether pretrained representations generalize across animals, sessions, and tasks while preserving behaviorally meaningful structure [13, 47, 48].

#### TS1: Tasks.

This task suite includes eight behavioral decoding tasks derived from the Brainwide Map decision-making task [34]. For each decoding task, the target is a 1 second window sampled around key trial events (i.e., stimulus onset, movement onset, feedback time) to ensure that there is both meaningful signal to decode the variable of interest and that we extract windows that are not confounded with other effects of movement (see Figure A8). In particular, we consider five *frame-level regression* tasks capturing continuous motor outputs: licking rate, whisker pad motion energy, wheel speed, left paw speed, and right paw speed (where wheel speed denotes the magnitude of angular velocity and paw speed the magnitude of 2D velocity); and three *sequence-level classification* tasks: stimulus contrast, choice, and reward. All of the continuous target variables are resampled at 50 Hz (details in Appendix B) and defined at each time point within the target window, producing a sequence-to-sequence prediction problem. See more details in Appendix D.1.

#### TS1: Evaluation Setup.

At evaluation, each session (from an animal unseen during training) is split across time such that the first 40% of trials fall in the train segment, the next 20% are in validation, and the remaining 40% are in test. This temporal split serves two purposes: it eliminates autocorrelation leakage between segments, and it reflects a realistic deployment scenario in which a small portion of data is used to calibrate the model before generating predictions on the remainder. This setting is particularly challenging due to the long duration of recording sessions (~1.4h average over evaluation sessions), hence models must learn to deal with nonstationarities and shifting probes which can affect the quality of spike sorting. Final metrics are computed on the standardized test split. The train and validation splits are provided as a reference protocol, and we adopt supervised finetuning on all available data for our baseline models (see Appendix G for finetuning details of individual models). However, the benchmark is designed to support alternative evaluation regimes, including few-shot and zero-shot transfer, enabling future work to explore different adaptation strategies. We measure performance using *R*^2^ for regression targets, balanced accuracy for classification targets, and *D*^2^ under a Poisson model for Licking Rate (rate variable).

#### TS1: Baselines.

We evaluate several pretrained models on TS1. We consider models to fall under the TSS setting if they pretrain on any of the decoding tasks, whereas TSU models utilize other forms of supervision or self-supervision during pretraining. In our framework, pretraining methods should produce a *single model* that can be applied downstream on all tasks in the suite. Hence, TSS models are naturally multi-task or otherwise assume cross-task transfer is achievable within the decoding targets; for this setting, we use POYO+ [15] and the multi-task version of POSSM [16]. For TSU models, we use NDT-Stitch [14, 17] and MtM [17] which both perform masked neural prediction. Note that MtM also uses anatomical information in its masking scheme. In addition, we benchmark several representative architectures on a single-session, single-task basis with extensive hyperparameter tuning. These single-session baselines establish a strong reference point for the pretrained models on individual decoding tasks, and provide a basis for comparing architectures in a controlled setting. For these baselines, we use Linear, MLP, CNN [49], GRU [50], CEBRA [51], POYO [13], and a supervised version of NDT [52]. See more details in Appendix F.

### 3.3 Task Suite 2: Neural Activity Prediction

#### TS2: Motivation.

Neural systems operate through dynamic interactions across neurons, where patterns of activity propagate, transform, and ultimately give rise to behavior. Modeling these dynamics requires predicting how different neurons or brain regions shape each other’s responses, making neural activity prediction a fundamental tool for capturing the temporal structure of population-level computation [31, 45]. By learning to predict neural activity, models can reveal the dependencies and interactions that govern circuit function, providing a direct lens on how information flows through the brain [53, 54]. Neural activity prediction thus serves as a testbed for *in silico* investigation of circuit mechanisms, enabling the construction of surrogate dynamical systems and brain-scale simulators [19, 55] to probe hypotheses about circuit function. At the same time, generative modeling capabilities afforded by such prediction tasks support practical advances, including data augmentation to improve downstream analyses [56, 57], enhanced interpretability through the discovery of latent structure in neural dynamics (e.g., Latent Variable Modeling [31, 58]), and real-time, closed-loop interfaces that adapt to evolving neural states [59].

#### TS2: Tasks.

This task suite includes two neural activity prediction tasks designed to probe complementary aspects of neural representation along both spatial and temporal dimensions. First, to assess a model’s ability to extract features of population-level structure and inter-neuron relationships, we design a *co-smoothing* task [31] where a random subset of neurons are masked within the context window, and the model must reconstruct their activity from the remaining observed neurons (Figure 1). At test time, the masked neurons form a fixed held-out set, ensuring consistent evaluation across samples. Next, to probe how well a model can extract fine-grained temporal structure from the population, we design a *forecasting* task with the goal of predicting future neural activity from past observations. This task assesses whether learned representations capture temporal dynamics and support extrapolation beyond the observed context. At test time, the last 200ms of each sample window are masked and set as the target for models to reconstruct. See more details in Appendix D.2.

#### TS2: Evaluation Setup.

We find the causal train/validation/test splits of TS1 to be especially difficult for neural activity prediction in TS2, limiting our ability to differentiate models. A possible explanation is that non-stationarity in neural recordings makes neuron identity unreliable across long timescales, so predicting the activity of a fixed, indexed set of neurons becomes ill-defined (see Appendix I.2). Hence, in TS2, we adopt a split structure consisting of interleaved 5-minute temporal blocks, with 40% of the session in train, 20% in validation, and 40% in test. This block structure reduces autocorrelation leakage that would arise from fully randomized splits while ensuring that each subset is large enough to contain representative samples of neural dynamics (see Appendix D.2 for more details). In both tasks, we evaluate performance using the fraction of deviance explained (*D*^2^) under a Poisson observation model and bits-per-spike (bps) [31]. *D*^2^ can also be termed Deviance Fraction Explained (DFE) as in [54]. See more details on these metrics in Appendix E.

#### TS2: Baselines.

In TS2, MtM [17] serves as a representative TSS model since it explicitly trains on masked neural prediction objectives related to co-smoothing and forecasting. We also evaluate NDT-Stitch [14, 17], which pretrains using temporal masked prediction but in a non-causal setting, making it fall under TSU even with respect to forecasting. In addition, we include single-session baselines using a standard autoencoder (AE) [60], LFADS [45], and NDT [52]. Following the Neural Latents Benchmark [31], these models are treated as latent variable models [58]: models are fit to training data to learn population representations, which are then evaluated on co-smoothing and forecasting tasks. While TS2 also supports direct optimization on the downstream tasks themselves, we focus on representation quality as the primary evaluation objective. See Appendix F for additional details. We also test simple statistical baselines that use training-set neural activity statistics, providing reference floors for how much co-smoothing and forecasting performance can be explained without learning a representation, and include these results in Appendix I.2.1.

### 3.4 Task Suite 3: Neuron Identity Prediction

#### TS3: Motivation.

A neuron’s identity, including its anatomical location and cell type, strongly shapes its activity patterns across behaviors and cognitive states [4]. Experimentally, identifying these properties typically relies on probe localization through histology and expert annotation [61]. However, these procedures are expensive, cannot be performed in vivo, and are subject to inter-rater variability [62], motivating the need for automated approaches that can infer neuronal identity directly from neural activity.

From a computational perspective, solving this task requires learning representations that capture biologically meaningful latent structure from neural signals alone. Prior work has shown that anatomical region and cell type can, to some extent, be decoded from activity patterns [63–65]. However, the Brainwide Map dataset [8] presents a substantially more challenging setting due to its broad anatomical coverage, large inter-animal variability, and diversity of recording conditions.

Motivated by these challenges, TS3 evaluates whether models recover intrinsic and biologically grounded structure from neural activity. Because brain regions differ in their connectivity, functional roles, cell type composition, and population statistics, they induce consistent signatures in neural dynamics that persist across animals and experimental conditions [66, 67]. We therefore evaluate brain region identification in a zero-shot transfer setting to test two complementary goals: whether learned representations uncover anatomical structure without explicit supervision, and whether they support practical in vivo localization in previously unseen animals.

#### TS3: Tasks.

Given neural activity from individual units, the task is to predict the associated anatomical region label. We align all probes to the Allen Common Coordinate Framework (CCF) [68] and use Cosmos-level annotations from the IBL anatomical atlas [8], resulting in a 10-class classification problem spanning coarse but functionally meaningful regions such as *Isocortex*, *Hippocampus*, and *Cerebellum*. We evaluate both the *single-unit* setting, where neurons are classified independently, and the *multi-unit* setting, where predictions are informed by neighboring units on the same probe. All evaluations are performed on held-out animals in a zero-shot setting, requiring generalization across recording sessions, probe placements, and inter-animal variability without using region labels from evaluation sessions during training. See Appendix D.3 for details.

#### TS3: Evaluation Setup.

We consider two across-animal evaluation settings designed to probe different aspects of generalization. The first is the *Transductive Zero-Shot* setting, where models are adapted on held-out animals using some form of supervision external to the brain region labels (e.g., behavioral labels as in POYO+), allowing them to adjust representations to the new domain before evaluation. This setting reflects realistic scenarios where some data from a new subject is available for adaptation. The second is the *Inductive Zero-Shot* setting, where pretrained models are evaluated on entirely unseen animals and probe configurations without any adaptation, providing a stringent test of whether learned representations capture invariant features of neural identity. Performance is measured using macro-averaged F1 score to account for class imbalance.

#### TS3: Baselines.

We evaluate methods for learning unit-level representations, including NEMO [64] and NuCLR [63], by pretraining them using their respective training objectives. Since these models are capable of generating embeddings for a new neural population via an inference step, they are representative baselines for the Inductive setting. In addition, we provide a simple baseline for the inductive setting using ISI features directly as embeddings. We also use LOLCAT from [65, 66] which is directly trained on the brain region classification task, without any pretraining. Since LOLCAT extracts features directly using the brain region classification task for supervision, it represents a TSS baseline for TS3, whereas NEMO and NuCLR fall under TSU. Finally, we evaluate unit-level embeddings from models originally pretrained for TS1 (POYO+ [15], POSSM [16]) and TS2 (NDT-Stitch [14, 17] and MtM [17]) as representative models for the Transductive setting. These models are finetuned on eval sessions using their pretraining objectives, as they require gradients to generate embeddings on the new neural population. See more details in Appendix F.

## 4 Benchmark Results

### 4.1 TS1 Results

In Table 1, we compare behavior decoding performance across pretrained models on both frame-level and sequence-level tasks. Unsurprisingly, TSS models pretrained directly on the decoding tasks (green) generally outperform TSU models pretrained on a different objective (magenta). However, across-task transfer remains surprisingly effective on frame-level tasks such as whisker and right paw, and sequence-level tasks such as reward and choice decoding. This suggests that pretrained representations capture latent neural structure that generalizes beyond their original supervision.

We also compare with single-session models to benchmark performance against representative architectures, though these require extensive tuning. Notably, POYO and NDT are both improved by their pretrained counterparts: POYO improves in average rank over tasks from 4.80 → 2.53 with POYO+, and NDT improves from 6.80 → 3.58 with NDT-Stitch (ranking procedure detailed in Appendix E.4). Among the single-session baselines, recurrent and convolutional models achieve strong performance across most tasks, particularly for high-signal variables such as licking and whisker motion. Nonetheless, POYO+ achieves the highest average rank among our baselines, and POSSM the second highest. Together, these results indicate that pretraining results in representations that transfer well across animals and decoding objectives.

### 4.2 TS2 Results

In Table 2, we evaluate neural activity prediction using both co-smoothing and forecasting tasks. Across all metrics, pretrained models outperform the single-session baselines, demonstrating that large-scale pretraining improves the ability to capture structured neural dynamics. In particular, NDT-Stitch achieves the strongest forecasting performance, while MtM performs best on co-smoothing, suggesting that different pretraining objectives emphasize complementary aspects of neural dynamics. Interestingly, NDT-Stitch is effective on forecasting despite being pretrained using a non-causal masked prediction objective, suggesting that representations learned through temporal reconstruction transfer well even with shift in the task distribution. Conversely, MtM performs particularly well on co-smoothing, likely due to the fact that for most training steps it employs a spatial masking strategy, hence a closer alignment to the downstream objective.

### 4.3 TS3 Results

In Table 3, we evaluate brain region identification across held-out animals in both transductive and inductive settings. Across all evaluation regimes, methods specifically designed to learn neuron-level representations substantially outperform models that optimize for a different objective, such as behavioral decoding or neural prediction. In particular, NuCLR achieves the strongest performance across both single-unit and multi-unit settings, reaching a macro-F1 of 0.654 in the multi-unit linear probe setting. NEMO also performs strongly with a peak macro-F1 of 0.605 with a multi-unit MLP probe. Both of these models focused on extracting invariant features of neuron identity. On the other hand, transductive methods such as POYO+, POSSM, NDT-Stitch, and MtM achieve substantially lower performance, suggesting that representations optimized for other objectives do not automatically organize around anatomical structure. Nevertheless, NDT-Stitch and MtM outperform POYO+ and POSSM, indicating that masked neural prediction may preserve more region-specific structure than behaviorally-aligned decoding objectives.

We also observe consistent improvements from single-unit to multi-unit evaluation, suggesting that neighboring neurons contain complementary information that improves localization accuracy. Interestingly, LOLCAT underperforms compared to some TSU baselines despite optimizing its latents for the downstream task. Overall, these results demonstrate that anatomical structure can emerge from neural activity representations in a zero-shot setting, while also highlighting the importance of pretraining objectives that explicitly encourage stable neuron-level representations across animals.

## 5 Discussion

This benchmark evaluates model generalization across three complementary axes: behavior, dynamics, and anatomy, providing a unified view of how large-scale models pretrained on neural data transfer across animals and downstream tasks. Across these axes, we observe a consistent and encouraging trend: pretrained models can act as strong generalist representations that rapidly adapt to new animals and recording conditions. At the same time, performance differences across task suites reveal important distinctions in what current pretraining objectives capture most effectively and where gaps still remain.

In TS1, pretrained behavioral models such as POYO+ and POSSM substantially outperform self-supervised approaches on downstream decoding tasks, suggesting that behaviorally-aligned objectives remain highly beneficial for extracting task-relevant neural structure. In TS2, pretraining consistently improves co-smoothing and forecasting performance relative to single-session latent variable baselines, demonstrating the value of large-scale data for modeling neural population dynamics. In TS3, we find that zero-shot brain region decoding across animals is possible, with self-supervised methods such as NuCLR and NEMO achieving strong performance without adaptation to evaluation animals. Together, these results suggest that pretrained models can uncover structure at scale that generalizes across individuals and probe locations, while also highlighting important differences in what current pretraining objectives capture most effectively. In particular, the gap between supervised and self-supervised approaches in TS1 highlights opportunities for SSL methods to better align their representations with behavior, while the strong performance of neuron-level SSL methods in TS3 suggests that preserving stable unit identity may be important for across-animal transfer.

More broadly, our results suggest that generalist neural models may help amortize the substantial cost of training and tuning models for individual sessions and tasks. Achieving strong single-session performance often requires extensive hyperparameter optimization, whereas pretrained models can be adapted to new animals and tasks using a largely standardized fine-tuning setup (see Appendix H for a breakdown of compute requirements per model, where we see single-session tuning is substantially more compute-intensive). Across our experiments, pretrained models were finetuned using minimal task-specific tuning, yet achieved competitive or superior performance across all settings. This suggests that large-scale pretraining not only improves transfer, but may also reduce the engineering burden required to deploy neural models across diverse datasets and experimental conditions. Still, important gaps remain in the ability of current models to transfer across new sessions and neural populations. For example, pretrained behavioral decoding and neural dynamics models still require target-session calibration to learn parameters specific to the new recording population, such as unit and session embeddings or session-specific read-in/read-out stitchers. Developing models for these tasks that require minimal or zero-shot calibration is a natural direction for future work that BrainWideBench is well suited to evaluate.

These conclusions should be interpreted in light of several limitations of the current benchmark. While BrainWideBench tests generalization across animals, brain regions, and downstream tasks, evaluation is limited to a single species (mouse), recording modality (Neuropixels electrophysiology), and behavioral paradigm (visually-guided decision-making). Furthermore, the held-out set was chosen to keep evaluation feasible while preserving broad anatomical coverage, but the result is a modest cohort of 13 animals. Thus, rankings are more reliable in aggregate than in fine-grained comparison: broad groupings of models hold up consistently across resampled splits, but rankings within a group are more sensitive to which animals are held out and should not be read as statistically distinguishable pointwise comparisons. Similarly, the categorical labels used throughout the benchmark (TSS versus TSU, full finetuning versus few-shot versus zero-shot, transductive versus inductive) organize model comparisons but do not fully capture the design choices and inductive biases built into each. Bringing disparate model families onto common ground for evaluation is precisely the benchmark’s aim, but these categories remain an imperfect proxy; model performance is therefore best read in aggregate across suites and with attention to each model’s underlying design choices, rather than as isolated, pairwise rankings. Within these constraints, the benchmark is best used to diagnose where current approaches generalize and where they fall short.

BrainWideBench provides a framework for systematically measuring progress toward general-purpose models of neural activity. By absorbing the substantial cost of baseline training, dataset curation and standardization into a single, fully documented evaluation suite, BrainWideBench allows researchers to focus on modeling and scientific questions, accelerating progress toward models that are genuinely useful for neuroscience.

## Supplementary Material

1

## Figures and Tables

**Table 1:** Task Suite 1: Behavior decoding performance. Single-session models are trained and tested on trials from the same session. Pretrained models are finetuned on the new sessions. Sequence-level tasks are reported as Balanced Accuracy (%); frame-level task performance is reported as *R*^2^, except for Licks, which is an event stream, where we report *D*^2^ instead. All metrics are reported as the average over evaluation sessions (±SEM over 5 finetuning seeds). Rankings incorporate statistical significance, see Appendix E.4 for more details. Green shading denotes a TSS baseline, while magenta denotes TSU. No shading denotes a single session baseline tuned and tested on the same session.

		Frame-level Tasks					Sequence-level Tasks			Avg
	Method	Licks (*D*^2^)	Whisker (*R*^2^)	Wheel (*R*^2^)	RPaw (*R*^2^)	LPaw (*R*^2^)	Reward (Acc)	Choice (Acc)	Contrast (Acc)	Rank
**Single-Session**	Linear	0.151 ± 0.000	0.175 ± 0.000	0.219 ± 0.002	0.091 ± 0.000	0.084 ± 0.001	0.813 ± 0.004	0.614 ± 0.004	0.219 ± 0.001	7.73
	MLP	0.348 ± 0.001	0.268 ± 0.002	0.329 ± 0.001	0.157 ± 0.001	0.135 ± 0.001	0.877 ± 0.006	0.636 ± 0.004	0.224 ± 0.002	5.46
	GRU	0.510 ± 0.002	0.358 ± 0.002	0.300 ± 0.006	0.214 ± 0.002	0.176 ± 0.002	0.885 ± 0.005	0.644 ± 0.007	0.214 ± 0.001	3.96
	CNN	0.549 ± 0.008	0.389 ± 0.004	0.330 ± 0.004	0.225 ± 0.004	0.194 ± 0.002	0.887 ± 0.004	0.621 ± 0.002	0.205 ± 0.002	3.74
	CEBRA	0.327 ± 0.003	0.280 ± 0.003	0.200 ± 0.002	0.164 ± 0.004	0.121 ± 0.002	0.780 ± 0.008	0.521 ± 0.002	0.198 ± 0.001	7.44
	NDT	0.314 ± 0.001	0.303 ± 0.002	0.212 ± 0.003	0.163 ± 0.002	0.127 ± 0.001	0.871 ± 0.005	0.616 ± 0.003	0.208 ± 0.003	6.80
	POYO	0.559 ± 0.002	0.291 ± 0.004	0.300 ± 0.003	0.195 ± 0.002	0.136 ± 0.002	0.882 ± 0.002	0.630 ± 0.005	0.224 ± 0.002	4.80
**Pretrain**	POYO+	0.678 ± 0.003	0.380 ± 0.003	0.362 ± 0.001	0.249 ± 0.002	0.200 ± 0.000	0.898 ± 0.003	0.689 ± 0.003	0.267 ± 0.001	2.53
	POSSM	0.661 ± 0.002	0.351 ± 0.003	0.355 ± 0.002	0.237 ± 0.001	0.175 ± 0.002	0.887 ± 0.004	0.662 ± 0.005	0.220 ± 0.002	3.18
	NDT-Stitch	0.525 ± 0.002	0.384 ± 0.003	0.310 ± 0.002	0.219 ± 0.005	0.172 ± 0.003	0.863 ± 0.006	0.674 ± 0.001	0.229 ± 0.001	3.58
	MtM	0.469 ± 0.004	0.362 ± 0.004	0.323 ± 0.006	0.224 ± 0.003	0.167 ± 0.003	0.874 ± 0.003	0.646 ± 0.004	0.213 ± 0.002	4.05

**Table 2:** Task Suite 2: Co-smoothing and forecasting performance. Model performance for both tasks is reported in terms of *D*^2^ and bps. All metrics are reported as the average over evaluation sessions (± SEM over 5 finetuning seeds). Rankings incorporate statistical significance, see Appendix E.4 for more details. Green shading denotes a TSS baseline, while magenta denotes TSU. No shading denotes a single session baseline tuned and tested on the same session.

	Method	Co-smoothing		Forecasting		Avg
		*D* ^2^	bps	*D* ^2^	bps	Rank
**SS**	Autoencoder	0.088 ± 0.000	0.200 ± 0.001	0.037 ± 0.001	0.065 ± 0.001	4.81
	LFADS	0.176 ± 0.000	0.421 ± 0.001	0.144 ± 0.000	0.325 ± 0.001	2.34
	NDT	0.132 ± 0.001	0.304 ± 0.003	0.158 ± 0.000	0.343 ± 0.001	2.79
**Pre**	NDT-Stitch	0.157 ± 0.001	0.369 ± 0.002	0.177 ± 0.000	0.384 ± 0.000	1.64
	MtM	0.191 ± 0.001	0.459 ± 0.002	0.131 ± 0.001	0.288 ± 0.001	2.57

**Table 3:** Task Suite 3: Brain region prediction performance. Results are reported as the macro-average F1 score (± SEM over 5 seeds). For learned embeddings, seeds parameterize embedding generation, i.e., through target-session calibration (finetuning) for transductive models, and through pretraining or supervised training for inductive models. Methods are evaluated at both Single Unit and Multi-Unit levels across Linear and MLP probes. All models are evaluated in zero-shot transfer with respect to the region labels after pretraining. Green shading denotes a TSS baseline, while magenta denotes TSU. We omit shading for the ISI baseline since its embeddings are not trained, and omit SEM on its linear probe since it is fully deterministic.

		Single Unit (F1)		Multi-Unit (F1)	
Regime	Method	Linear Probe	MLP Probe	Linear Probe	MLP Probe
	POYO+	0.099 ± 0.003	0.120 ± 0.005	0.072 ± 0.001	0.132 ± 0.008
**Transductive**	POSSM	0.103 ± 0.002	0.123 ± 0.003	0.104 ± 0.004	0.133 ± 0.006
	NDT-Stitch	0.136 ± 0.007	0.167 ± 0.007	0.155 ± 0.011	0.187 ± 0.008
	MtM	0.137 ± 0.004	0.140 ± 0.006	0.159 ± 0.009	0.158 ± 0.007
	ISI Baseline	0.263	0.380 ± 0.002	0.355	0.519 ± 0.006
**Inductive**	LOLCAT	0.359 ± 0.003	–	0.473 ± 0.004	–
	NEMO	0.447 ± 0.003	0.471 ± 0.002	0.579 ± 0.007	0.605 ± 0.004
	NuCLR	0.616 ± 0.006	0.626 ± 0.005	0.654 ± 0.010	0.645 ± 0.003

## Footnotes

2 Data is processed into the standardized benchmark format using torch_brain and released for download here. We also release the processing pipeline in our codebase for reproducibility.

## References

[1] Jun J. J., Steinmetz N. A., Siegle J. H., Denman D. J., Bauza M., Barbarits B., Lee A. K., Anastassiou C. A., Andrei A., Aydın Ç., , “Fully integrated silicon probes for high-density recording of neural activity,” Nature, vol. 551, no. 7679, pp. 232–236, 2017. 10.1038/nature24636 PMC5955206 29120427

[2] Steinmetz N. A., Aydin C., Lebedeva A., Okun M., Pachitariu M., Bauza M., Beau M., Bhagat J., Böhm C., Broux M., , “Neuropixels 2.0: A miniaturized high-density probe for stable, long-term brain recordings,” Science, vol. 372, no. 6539, p. eabf4588, 2021. 10.1126/science.abf4588 PMC8244810 33859006

[3] Ye Z., Shelton A. M., Shaker J. R., Boussard J., Colonell J., Birman D., Manavi S., Chen S., Windolf C., Hurwitz C., , “Ultra-high-density neuropixels probes improve detection and identification in neuronal recordings,” Neuron, vol. 113, no. 23, pp. 3966–3982, 2025. 10.1016/j.neuron.2025.08.030 PMC12981004 41033305

[4] Steinmetz N. A., Zatka-Haas P., Carandini M., and Harris K. D., “Distributed coding of choice, action and engagement across the mouse brain,” Nature, vol. 576, no. 7786, pp. 266–273, 2019. 10.1038/s41586-019-1787-x PMC6913580 31776518

[5] Ottenheimer D. J., Hjort M. M., Bowen A. J., Steinmetz N. A., and Stuber G. D., “A stable, distributed code for cue value in mouse cortex during reward learning,” Elife, vol. 12, p. RP84604, 2023. 10.7554/eLife.84604 PMC10328514 37382590

[6] Chen S., Liu Y., Wang Z. A., Colonell J., Liu L. D., Hou H., Tien N.-W., Wang T., Harris T., Druckmann S., , “Brain-wide neural activity underlying memory-guided movement,” Cell, vol. 187, no. 3, pp. 676–691, 2024. 10.1016/j.cell.2023.12.035 PMC11492138 38306983

[7] Khilkevich A., Lohse M., Low R., Orsolic I., Bozic T., Windmill P., and Mrsic-Flogel T. D., “Brain-wide dynamics linking sensation to action during decision-making,” Nature, vol. 634, no. 8035, pp. 890–900, 2024. 10.1038/s41586-024-07908-w PMC11499283 39261727

[8] IBL, Benson B., Benson J., Birman D., Bonacchi N., Carandini M., Catarino J. A., Chapuis G. A., Churchland A. K., Dan Y., Dayan P., DeWitt E. E., Engel T. A., Fabbri M., Faulkner M., Fiete I. R., Findling C., Freitas-Silva L., Gerçek B., Harris K. D., Häusser M., Hofer S. B., Hu F., Hubert F., Huntenburg J. M., Khanal A., Krasniak C., Langdon C., Lau P. Y. P., Mainen Z. F., Meijer G. T., Miska N. J., Mrsic-Flogel T. D., Noel J.-P., Nylund K., Pan-Vazquez A., Pouget A., Rossant C., Roth N., Schaeffer R., Schartner M., Shi Y., Socha K. Z., Steinmetz N. A., Svoboda K., Urai A. E., Wells M. J., West S. J., Whiteway M. R., Winter O., and Witten I. B., “A brain-wide map of neural activity during complex behaviour,” Nature, vol. 645, no. 8079, pp. 177–191, 2025. 10.1038/s41586-025-09235-0 PMC12408349 40903598

[9] Findling C., Hubert F., IBL, Acerbi L., Benson B., Benson J., Birman D., Bonacchi N., Buchanan E. K., Bruijns S., , “Brain-wide representations of prior information in mouse decision-making,” Nature, vol. 645, no. 8079, pp. 192–200, 2025. 10.1038/s41586-025-09226-1 PMC12408363 40903597

[10] Stringer C. and Pachitariu M., “Analysis methods for large-scale neuronal recordings,” Science, vol. 386, no. 6722, p. eadp7429, 2024. 10.1126/science.adp7429 39509504

[11] Dyer E. and Richards B., “Accepting “the bitter lesson” and embracing the brain’s complexity,” SPECTRUM, 2025.

[12] Bommasani R., Hudson D. A., Adeli E., Altman R., Arora S., von Arx S., Bernstein M. S., Bohg J., Bosselut A., Brunskill E., , “On the opportunities and risks of foundation models,” arXiv *preprint arXiv:2108.07258*, 2021.

[13] Azabou M., Arora V., Ganesh V., Mao X., Nachimuthu S., Mendelson M., Richards B., Perich M., Lajoie G., and Dyer E., “A unified, scalable framework for neural population decoding,” Advances in Neural Information Processing Systems, vol. 36, 2024.

[14] Ye J., Collinger J., Wehbe L., and Gaunt R., “Neural data transformer 2: multi-context pretraining for neural spiking activity,” Advances in Neural Information Processing Systems, vol. 36, pp. 80352–80374, 2023.

[15] Azabou M., Pan K. X., Arora V., Knight I. J., Dyer E. L., and Richards B. A., “Multi-session, multi-task neural decoding from distinct cell-types and brain regions,” in The Thirteenth International Conference on Learning Representations, 2025.

[16] Ryoo A. H.-W., Krishna N. H., Mao X., Azabou M., Dyer E. L., Perich M. G., and Lajoie G., “Generalizable, real-time neural decoding with hybrid state-space models,” in The Thirty-ninth Annual Conference on Neural Information Processing Systems, 2025.

[17] Zhang Y., Wang Y., Jiménez-Benetó D. M., Wang Z., Azabou M., Richards B., Tung R., Winter O., Dyer E., Paninski L., , “Towards a “universal translator” for neural dynamics at single-cell, single-spike resolution,” Advances in Neural Information Processing Systems, vol. 37, pp. 80495–80521, 2024. PMC12228527 40620317

[18] Zhang Y., Wang Y., Azabou M., Andre A., Wang Z., Lyu H., Dyer E. L., Paninski L., Hurwitz C. L., , “Neural encoding and decoding at scale,” in Forty-second International Conference on Machine Learning, 2025.

[19] Wang E. Y., Fahey P. G., Ding Z., Papadopoulos S., Ponder K., Weis M. A., Chang A., Muhammad T., Patel S., Ding Z., , “Foundation model of neural activity predicts response to new stimulus types,” Nature, vol. 640, no. 8058, pp. 470–477, 2025. 10.1038/s41586-025-08829-y PMC11981942 40205215

[20] Vermani A., Nassar J., Jeon H., Dowling M., and Park I. M., “Meta-dynamical state space models for integrative neural data analysis,” in The Thirteenth International Conference on Learning Representations, 2025.

[21] Radford A., Kim J. W., Hallacy C., Ramesh A., Goh G., Agarwal S., Sastry G., Askell A., Mishkin P., Clark J., , “Learning transferable visual models from natural language supervision,” in International conference on machine learning, pp. 8748–8763, PmLR, 2021.

[22] Hoffmann J., Borgeaud S., Mensch A., Buchatskaya E., Cai T., Rutherford E., de Las Casas D., Hendricks L. A., Welbl J., Clark A., Hennigan T., Noland E., Millican K., van den Driessche G., Damoc B., Guy A., Osindero S., Simonyan K., Elsen E., Vinyals O., Rae J., and Sifre L., “An empirical analysis of compute-optimal large language model training,” in Advances in Neural Information Processing Systems, vol. 35, pp. 30016–30030, Curran Associates, Inc., 2022.

[23] Donoho D., “50 years of data science,” Journal of computational and graphical statistics, vol. 26, no. 4, pp. 745–766, 2017.

[24] Zellers R., Holtzman A., Bisk Y., Farhadi A., and Choi Y., “Hellaswag: Can a machine really finish your sentence?,” in Proceedings of the 57th annual meeting of the association for computational linguistics, pp. 4791–4800, 2019.

[25] Wang A., Pruksachatkun Y., Nangia N., Singh A., Michael J., Hill F., Levy O., and Bowman S., “Superglue: A stickier benchmark for general-purpose language understanding systems,” Advances in neural information processing systems, vol. 32, 2019.

[26] Hendrycks D., Burns C., Basart S., Zou A., Mazeika M., Song D., and Steinhardt J., “Measuring massive multitask language understanding,” in International Conference on Learning Representations, 2021.

[27] Cobbe K., Kosaraju V., Bavarian M., Chen M., Jun H., Kaiser L., Plappert M., Tworek J., Hilton J., Nakano R., , “Training verifiers to solve math word problems,” arXiv *preprint arXiv:2110.14168*, 2021.

[28] Chen M., Tworek J., Jun H., Yuan Q., Pinto H. P. D. O., Kaplan J., Edwards H., Burda Y., Joseph N., Brockman G., , “Evaluating large language models trained on code,” arXiv *preprint arXiv:2107.03374*, 2021.

[29] Notin P., Kollasch A., Ritter D., Van Niekerk L., Paul S., Spinner H., Rollins N., Shaw A., Orenbuch R., Weitzman R., , “Proteingym: Large-scale benchmarks for protein fitness prediction and design,” Advances in Neural Information Processing Systems, vol. 36, pp. 64331–64379, 2023.

[30] Dunn A., Wang Q., Ganose A., Dopp D., and Jain A., “Benchmarking materials property prediction methods: the matbench test set and automatminer reference algorithm,” npj Computational Materials, vol. 6, no. 1, p. 138, 2020.

[31] Pei F. C., Ye J., Zoltowski D. M., Wu A., Chowdhury R. H., Sohn H., O’Doherty J. E., Shenoy K. V., Kaufman M., Churchland M. M., Jazayeri M., Miller L. E., Pillow J. W., Park I. M., Dyer E. L., and Pandarinath C., “Neural latents benchmark ‘21: Evaluating latent variable models of neural population activity,” in Thirty-fifth Conference on Neural Information Processing Systems Datasets and Benchmarks Track (Round 2), 2021.

[32] Karpowicz B. M., Ye J., Fan C., Tostado-Marcos P., Rizzoglio F., Washington C. B., Scodeler T., de Lucena D. S., Nason-Tomaszewski S. R., Mender M., Ma X., Arneodo E. M., Hochberg L., Chestek C., Henderson J. M., Gentner T. Q., Gilja V., Miller L. E., Rouse A. G., Gaunt R., Collinger J. L., and Pandarinath C., “Few-shot algorithms for consistent neural decoding (FALCON) benchmark,” in The Thirty-eight Conference on Neural Information Processing Systems Datasets and Benchmarks Track, 2024.

[33] Willeke K. F., Fahey P. G., Bashiri M., Pede L., Burg M. F., Blessing C., Cadena S. A., Ding Z., Lurz K.-K., Ponder K., , “The sensorium competition on predicting large-scale mouse primary visual cortex activity,” arXiv *preprint arXiv:2206.08666*, 2022.

[34] IBL, Aguillon-Rodriguez V., Angelaki D., Bayer H., Bonacchi N., Carandini M., Cazettes F., Chapuis G., Churchland A. K., Dan Y., Dewitt E., Faulkner M., Forrest H., Haetzel L., Häusser M., Hofer S. B., Hu F., Khanal A., Krasniak C., Laranjeira I., Mainen Z. F., Meijer G., Miska N. J., Mrsic-Flogel T. D., Murakami M., Noel J.-P., Pan-Vazquez A., Rossant C., Sanders J., Socha K., Terry R., Urai A. E., Vergara H., Wells M., Wilson C. J., Witten I. B., Wool L. E., and Zador A. M., “Standardized and reproducible measurement of decision-making in mice,” eLife, vol. 10, p. e63711, may 2021. 10.7554/eLife.63711 PMC8137147 34011433

[35] Abbott L. F., Angelaki D. E., Carandini M., Churchland A. K., Dan Y., Dayan P., Deneve S., Fiete I., Ganguli S., Harris K. D., , “An international laboratory for systems and computational neuroscience,” Neuron, vol. 96, no. 6, pp. 1213–1218, 2017. 10.1016/j.neuron.2017.12.013 PMC5752703 29268092

[36] IBL, Bonacchi N., Chapuis G. A., Churchland A. K., DeWitt E. E., Faulkner M., Harris K. D., Huntenburg J. M., Hunter M., Laranjeira I. C., , “A modular architecture for organizing, processing and sharing neurophysiology data,” Nature methods, vol. 20, no. 3, pp. 403–407, 2023. 10.1038/s41592-022-01742-6 PMC7614641 36864199

[37] IBL, Banga K., Benson J., Bhagat J., Biderman D., Birman D., Bonacchi N., Bruijns S. A., Buchanan K., Campbell R. A., , “Reproducibility of in vivo electrophysiological measurements in mice,” Elife, vol. 13, p. RP100840, 2025. 10.7554/eLife.100840 PMC12068871 40354112

[38] Biderman D., Whiteway M. R., Hurwitz C., Greenspan N., Lee R. S., Vishnubhotla A., Warren R., Pedraja F., Noone D., Schartner M. M., , “Lightning pose: improved animal pose estimation via semi-supervised learning, bayesian ensembling and cloud-native open-source tools,” Nature Methods, vol. 21, no. 7, pp. 1316–1328, 2024. 10.1038/s41592-024-02319-1 PMC12087009 38918605

[39] IBL, Boussard J., Chapuis G. A., Harris K. D., Langfield C., Paninski L., Rossant C., Roth N., Steinmetz N. A., Windolf C., and Winter O., “Spike sorting pipeline for the International Brain Laboratory,” figshare, 5 2022.

[40] IBL, Birman D., Bonacchi N., Buchanan K., Chapuis G., Huntenburg J., Meijer G., Paninski L., Schartner M., Svoboda K., Whiteway M., Wells M., and Winter O., “Video hardware and software for the international brain laboratory,” figshare, 2022.

[41] Cunningham J. P. and Yu B. M., “Dimensionality reduction for large-scale neural recordings,” Nature neuroscience, vol. 17, no. 11, pp. 1500–1509, 2014. 10.1038/nn.3776 PMC4433019 25151264

[42] Churchland M. M., Cunningham J. P., Kaufman M. T., Foster J. D., Nuyujukian P., Ryu S. I., and Shenoy K. V., “Neural population dynamics during reaching,” Nature, vol. 487, no. 7405, pp. 51–56, 2012. 10.1038/nature11129 PMC3393826 22722855

[43] Glaser J. I., Benjamin A. S., Chowdhury R. H., Perich M. G., Miller L. E., and Kording K. P., “Machine learning for neural decoding,” eneuro, vol. 7, no. 4, 2020. 10.1523/ENEURO.0506-19.2020 PMC7470933 32737181

[44] Paninski L., Pillow J., and Lewi J., “Statistical models for neural encoding, decoding, and optimal stimulus design,” Progress in brain research, vol. 165, pp. 493–507, 2007. 10.1016/S0079-6123(06)65031-0 17925266

[45] Pandarinath C., Ames K. C., Russo A. A., Farshchian A., Miller L. E., Dyer E. L., and Kao J. C., “Latent factors and dynamics in motor cortex and their application to brain–machine interfaces,” Journal of Neuroscience, vol. 38, no. 44, pp. 9390–9401, 2018. 10.1523/JNEUROSCI.1669-18.2018 PMC6209846 30381431

[46] Pandarinath C., Nuyujukian P., Blabe C. H., Sorice B. L., Saab J., Willett F. R., Hochberg L. R., Shenoy K. V., and Henderson J. M., “High performance communication by people with paralysis using an intracortical brain-computer interface,” elife, vol. 6, p. e18554, 2017. 10.7554/eLife.18554 PMC5319839 28220753

[47] Ye J., Rizzoglio F., Smoulder A., Mao H., Ma X., Marino P., Chowdhury R., Moore D., Blumenthal G., Hockeimer W., , “A generalist intracortical motor decoder,” bioRxiv, 2025.

[48] Mao X., Krishna N. H., Ryoo A. H.-W., Perich M. G., and Lajoie G., “Leveraging unlabelled data for generalizable neural population decoding,” 2026.

[49] Lea C., Vidal R., Reiter A., and Hager G. D., “Temporal convolutional networks: A unified approach to action segmentation,” in European conference on computer vision, pp. 47–54, Springer, 2016.

[50] Cho K., van Merriënboer B., Gulcehre C., Bahdanau D., Bougares F., Schwenk H., and Bengio Y., “Learning phrase representations using RNN encoder–decoder for statistical machine translation,” in Proceedings of the 2014 Conference on Empirical Methods in Natural Language Processing (EMNLP) (Moschitti A., Pang B., and Daelemans W., eds.), (Doha, Qatar), pp. 1724–1734, Association for Computational Linguistics, Oct. 2014.

[51] Schneider S., Lee J. H., and Mathis M. W., “Learnable latent embeddings for joint behavioural and neural analysis,” Nature, May 2023. 10.1038/s41586-023-06031-6 PMC10172131 37138088

[52] Ye J. and Pandarinath C., “Representation learning for neural population activity with neural data transformers,” arXiv *preprint arXiv:2108.01210*, 2021.

[53] Gokcen E., Jasper A. I., Semedo J. D., Zandvakili A., Kohn A., Machens C. K., and Yu B. M., “Disentangling the flow of signals between populations of neurons,” Nature Computational Science, vol. 2, no. 8, pp. 512–525, 2022. 10.1038/s43588-022-00282-5 PMC11442031 38177794

[54] Xia J., Zhang Y., Wang S., Allen G. I., Paninski L., Hurwitz C. L., and Miller K. D., “Inpainting the neural picture: Inferring unrecorded brain area dynamics from multi-animal datasets,” in The Thirty-ninth Annual Conference on Neural Information Processing Systems, 2026.

[55] Haspel G., Baker B., Beets I., Boyden E. S., Brown J., Church G., Cohen N., Colon-Ramos D., Dyer E., Fang-Yen C., , “The time is ripe to reverse engineer an entire nervous system: simulating behavior from neural interactions,” arXiv *preprint arXiv:2308.06578*, 2023.

[56] Kapoor J., Schulz A., Vetter J., Pei F. C., Gao R., and Macke J. H., “Latent diffusion for neural spiking data,” in The Thirty-eighth Annual Conference on Neural Information Processing Systems, 2024.

[57] McCart J. D., Sedler A. R., Versteeg C., Mifsud D., Rigotti-Thompson M., and Pandarinath C., “Diffusion-based generation of neural activity from disentangled latent codes,” 2024.

[58] Hurwitz C., Kudryashova N., Onken A., and Hennig M. H., “Building population models for large-scale neural recordings: Opportunities and pitfalls,” Current opinion in neurobiology, vol. 70, pp. 64–73, 2021. 10.1016/j.conb.2021.07.003 34411907

[59] Minai Y., Soldado-Magraner J., Yu B. M., and Smith M. A., “OMiSO: Adaptive optimization of state-dependent brain stimulation to shape neural population states,” in The Thirty-ninth Annual Conference on Neural Information Processing Systems, 2026.

[60] Hinton G. E. and Salakhutdinov R. R., “Reducing the dimensionality of data with neural networks,” Science, vol. 313, no. 5786, pp. 504–507, 2006. 10.1126/science.1127647 16873662

[61] Liu L. D., Chen S., Hou H., West S. J., Faulkner M., Laboratory I. B., Economo M. N., Li N., and Svoboda K., “Accurate localization of linear probe electrode arrays across multiple brains,” ENeuro, vol. 8, no. 6, pp. ENEURO–0241, 2021. 10.1523/ENEURO.0241-21.2021 PMC8597948 34697075

[62] Carey H., Pegios M., Martin L., Saleeba C., Turner A. J., Everett N. A., Bjerke I. E., Puchades M. A., Bjaalie J. G., and McMullan S., “Deepslice: rapid fully automatic registration of mouse brain imaging to a volumetric atlas,” Nature Communications, vol. 14, no. 1, p. 5884, 2023. 10.1038/s41467-023-41645-4 PMC10514056 37735467

[63] Arora V., Lachi D., Knight I. J., Azabou M., Richards B. A., Hurwitz C. L., Siegle J., and Dyer E. L., “Know thyself by knowing others: Learning neuron identity from population context,” in The Thirty-ninth Annual Conference on Neural Information Processing Systems, 2026.

[64] Yu H., Lyu H., Xu Y., Windolf C., Lee E. K., Yang F., Shelton A. M., Winter O., Laboratory I. B., Dyer E. L., Chandrasekaran C., Steinmetz N. A., Paninski L., and Hurwitz C. L., “In vivo cell-type and brain region classification via multimodal contrastive learning,” in The Thirteenth International Conference on Learning Representations, 2025.

[65] Schneider A., Azabou M., McDougall-Vigier L., Parks D. F., Ensley S., Bhaskaran-Nair K., Nowakowski T., Dyer E. L., and Hengen K. B., “Transcriptomic cell type structures in vivo neuronal activity across multiple timescales,” Cell reports, vol. 42, no. 4, 2023. 10.1016/j.celrep.2023.112318 PMC10539488 36995938

[66] Tolossa G. B., Schneider A. M., Dyer E., and Hengen K. B., “Neurons throughout the brain embed robust signatures of their anatomical location into spike trains,” Elife, vol. 13, p. RP101506, 2025. 10.7554/eLife.101506 PMC12204685 40577195

[67] Schneider A., Robust Embeddings of Genetics, Anatomy, and State Decoded from a Neuron’s Activity. PhD thesis, Washington University in St. Louis, 2025.

[68] Wang Q., Ding S.-L., Li Y., Royall J., Feng D., Lesnar P., Graddis N., Naeemi M., Facer B., Ho A., , “The allen mouse brain common coordinate framework: a 3d reference atlas,” Cell, vol. 181, no. 4, pp. 936–953, 2020. 10.1016/j.cell.2020.04.007 PMC8152789 32386544

[69] Weitzel D., Graves A., Albin S., Zhu H., Wuerthwein F., Tatineni M., Mishin D., Khoda E., Sada M., Smarr L., DeFanti T., and Graham J., “The national research platform: Stretched, multi-tenant, scientific kubernetes cluster,” in Practice and Experience in Advanced Research Computing 2025: The Power of Collaboration, PEARC ’25, (New York, NY, USA), Association for Computing Machinery, 2025.

[70] Laboratory I. B., “Data release - brainwide map - technical paper,” Figshare, 2022.

[71] Harris K. D., “Nonsense correlations in neuroscience,” biorxiv, pp. 2020–11, 2020.

[72] Bergstra J., Bardenet R., Bengio Y., and Kégl B., “Algorithms for hyper-parameter optimization,” Advances in neural information processing systems, vol. 24, 2011.

[73] Akiba T., Sano S., Yanase T., Ohta T., and Koyama M., “Optuna: A next-generation hyper-parameter optimization framework,” in Proceedings of the 25th ACM SIGKDD international conference on knowledge discovery & data mining, pp. 2623–2631, 2019.

[74] Cameron A. C. and Windmeijer F. A., “An r-squared measure of goodness of fit for some common nonlinear regression models,” Journal of econometrics, vol. 77, no. 2, pp. 329–342, 1997.

[75] McCullagh P., Nelder J. A., and McCullagh P., Generalized linear models, vol. 2. Chapman and hall London, 1989.

[76] Demšar J., “Statistical comparisons of classifiers over multiple data sets,” Journal of Machine learning research, vol. 7, no. Jan, pp. 1–30, 2006.

[77] Dror R., Baumer G., Shlomov S., and Reichart R., “The hitchhiker’s guide to testing statistical significance in natural language processing,” in Proceedings of the 56th annual meeting of the association for computational linguistics (volume 1: Long papers), pp. 1383–1392, 2018.

[78] Piepho H.-P., “An algorithm for a letter-based representation of all-pairwise comparisons,” Journal of Computational and Graphical Statistics, vol. 13, no. 2, pp. 456–466, 2004.

[79] Virtanen P., Gommers R., Oliphant T. E., Haberland M., Reddy T., Cournapeau D., Burovski E., Peterson P., Weckesser W., Bright J., , “Scipy 1.0: fundamental algorithms for scientific computing in python,” Nature methods, vol. 17, no. 3, pp. 261–272, 2020. 10.1038/s41592-019-0686-2 PMC7056644 32015543

[80] Sennrich R., Haddow B., and Birch A., “Neural machine translation of rare words with subword units,” in Proceedings of the 54th annual meeting of the association for computational linguistics (volume 1: long papers), pp. 1715–1725, 2016.

[81] Dosovitskiy A., Beyer L., Kolesnikov A., Weissenborn D., Zhai X., Unterthiner T., Dehghani M., Minderer M., Heigold G., Gelly S., , “An image is worth 16×16 words: Transformers for image recognition at scale,” arXiv *preprint arXiv:2010.11929*, 2020.

[82] Adrian E. D., “The impulses produced by sensory nerve endings: Part i,” The Journal of physiology, vol. 61, no. 1, p. 49, 1926. 10.1113/jphysiol.1926.sp002273 PMC1514809 16993776

[83] Le T. and Shlizerman E., “Stndt: Modeling neural population activity with spatiotemporal transformers,” Advances in Neural Information Processing Systems, vol. 35, pp. 17926–17939, 2022.

[84] Thorpe S. and Gautrais J., “Rank order coding,” in Computational Neuroscience: Trends in Research, 1998, pp. 113–118, Springer, 1998.

[85] Panzeri S., Brunel N., Logothetis N. K., and Kayser C., “Sensory neural codes using multiplexed temporal scales,” Trends in neurosciences, vol. 33, no. 3, pp. 111–120, 2010. 10.1016/j.tins.2009.12.001 20045201

[86] Perkel D. H., Gerstein G. L., and Moore G. P., “Neuronal spike trains and stochastic point processes: I. the single spike train,” Biophysical journal, vol. 7, no. 4, pp. 391–418, 1967. 10.1016/S0006-3495(67)86596-2 PMC1368068 4292791

[87] Dorval A. D., “Probability distributions of the logarithm of inter-spike intervals yield accurate entropy estimates from small datasets,” Journal of neuroscience methods, vol. 173, no. 1, pp. 129–139, 2008. 10.1016/j.jneumeth.2008.05.013 PMC2610469 18620755

[88] Gerstner W., Kistler W. M., Naud R., and Paninski L., Neuronal dynamics: From single neurons to networks and models of cognition. Cambridge University Press, 2014.

[89] Gold C., Henze D. A., Koch C., and Buzsaki G., “On the origin of the extracellular action potential waveform: a modeling study,” Journal of neurophysiology, vol. 95, no. 5, pp. 3113–3128, 2006. 10.1152/jn.00979.2005 16467426

[90] Elman J. L., “Finding structure in time,” Cognitive science, vol. 14, no. 2, pp. 179–211, 1990.

[91] Bengio Y., Simard P., and Frasconi P., “Learning long-term dependencies with gradient descent is difficult,” IEEE Transactions on Neural Networks, vol. 5, no. 2, pp. 157–166, 1994. 10.1109/72.279181 18267787

[92] Hochreiter S. and Schmidhuber J., “Long short-term memory,” Neural Computation, vol. 9, no. 8, pp. 1735–1780, 1997. 10.1162/neco.1997.9.8.1735 9377276

[93] Gu A., Goel K., and Re C., “Efficiently modeling long sequences with structured state spaces,” in International Conference on Learning Representations, 2022.

[94] Yu B. M., Cunningham J., Santhanam G., Ryu S., Shenoy K. V., and Sahani M., “Gaussian-process factor analysis for low-dimensional single-trial analysis of neural population activity,” in Advances in Neural Information Processing Systems (Koller D., Schuurmans D., Bengio Y., and Bottou L., eds.), vol. 21, Curran Associates, Inc., 2008.

[95] Macke J. H., Buesing L., Cunningham J., Yu B. M., Shenoy K. V., and Sahani M., “Empirical models of spiking in neural populations,” in Advances in Neural Information Processing Systems (Shawe-Taylor J., Zemel R., Bartlett P., Pereira F., and Weinberger K., eds.), vol. 24, Curran Associates, Inc., 2011.

[96] She Q. and Wu A., “Neural dynamics discovery via gaussian process recurrent neural networks,” in Proceedings of The 35th Uncertainty in Artificial Intelligence Conference (Adams R. P. and Gogate V., eds.), vol. 115 of *Proceedings of Machine Learning Research*, pp. 454–464, PMLR, 22–25 Jul 2020.

[97] Gu A. and Dao T., “Mamba: Linear-time sequence modeling with selective state spaces,” in First Conference on Language Modeling, 2024.

[98] Pandarinath C., O’Shea D. J., Collins J., Jozefowicz R., Stavisky S. D., Kao J. C., Trautmann E. M., Kaufman M. T., Ryu S. I., Hochberg L. R., , “Inferring single-trial neural population dynamics using sequential auto-encoders,” Nature methods, vol. 15, no. 10, pp. 805–815, 2018. 10.1038/s41592-018-0109-9 PMC6380887 30224673

[99] Keshtkaran M. R., Sedler A. R., Chowdhury R. H., Tandon R., Basrai D., Nguyen S. L., Sohn H., Jazayeri M., Miller L. E., and Pandarinath C., “A large-scale neural network training framework for generalized estimation of single-trial population dynamics,” Nature methods, vol. 19, no. 12, pp. 1572–1577, 2022. 10.1038/s41592-022-01675-0 PMC9825111 36443486

[100] Jaegle A., Borgeaud S., Alayrac J.-B., Doersch C., Ionescu C., Ding D., Koppula S., Zoran D., Brock A., Shelhamer E., , “Perceiver io: A general architecture for structured inputs & outputs,” arXiv *preprint arXiv:2107.14795*, 2021.

[101] Su J., Ahmed M., Lu Y., Pan S., Bo W., and Liu Y., “Roformer: Enhanced transformer with rotary position embedding,” Neurocomputing, vol. 568, p. 127063, 2024.

[102] de Vries S. E., Lecoq J. A., Buice M. A., Groblewski P. A., Ocker G. K., Oliver M., Feng D., Cain N., Ledochowitsch P., Millman D., , “A large-scale standardized physiological survey reveals functional organization of the mouse visual cortex,” Nature neuroscience, vol. 23, no. 1, pp. 138–151, 2020. 10.1038/s41593-019-0550-9 PMC6948932 31844315

[103] Devlin J., Chang M.-W., Lee K., and Toutanova K., “Bert: Pre-training of deep bidirectional transformers for language understanding,” in Proceedings of the 2019 conference of the North American chapter of the association for computational linguistics: human language technologies, volume 1 (long and short papers), pp. 4171–4186, 2019.

[104] Chen T., Kornblith S., Norouzi M., and Hinton G., “A simple framework for contrastive learning of visual representations,” 2020.

[105] He K., Chen X., Xie S., Li Y., Dollár P., and Girshick R., “Masked autoencoders are scalable vision learners,” in Proceedings of the IEEE/CVF conference on computer vision and pattern recognition, pp. 16000–16009, 2022.

[106] Loshchilov I. and Hutter F., “Decoupled weight decay regularization,” 2019.

[107] Smith L. N. and Topin N., “Super-convergence: Very fast training of neural networks using large learning rates,” 2018.

[108] He T., Zhang Z., Zhang H., Zhang Z., Xie J., and Li M., “Bag of tricks for image classification with convolutional neural networks,” in 2019 IEEE/CVF Conference on Computer Vision and Pattern Recognition (CVPR), pp. 558–567, IEEE, 2019.

[109] Jia X., Song S., He W., Wang Y., Rong H., Zhou F., Xie L., Guo Z., Yang Y., Yu L., , “Highly scalable deep learning training system with mixed-precision: Training imagenet in four minutes,” arXiv *preprint arXiv:1807.11205*, 2018.

[110] Huang X. S., Perez F., Ba J., and Volkovs M., “Improving transformer optimization through better initialization,” in Proceedings of the 37th International Conference on Machine Learning (D. H. III and Singh A., eds.), vol. 119 of *Proceedings of Machine Learning Research*, pp. 4475–4483, PMLR, 13–18 Jul 2020.

[111] Beau M., D’Agostino F., Lajko A., Martínez G., and Kostadinov D., “Neuropyxels: loading, processing and plotting neuropixels data in python,” Zenodo 10.5281/ZENODO, vol. 5509776, 2021.

[112] Beau M., Herzfeld D. J., Naveros F., Hemelt M. E., D’Agostino F., Oostland M., Sánchez-López A., Chung Y. Y., Maibach M., Stabb H. N., , “A deep-learning strategy to identify cell types across species from high-density extracellular recordings,” Cell, 2025. 10.1016/j.cell.2025.01.041 PMC12191827 40023155

[113] Hendrycks D. and Gimpel K., “Gaussian error linear units (gelus),” arXiv *preprint arXiv:1606.08415*, 2016.

[114] He K., Zhang X., Ren S., and Sun J., “Deep residual learning for image recognition,” in Proceedings of the IEEE conference on computer vision and pattern recognition, pp. 770–778, 2016.

[115] Berens P., Freeman J., Deneux T., Chenkov N., McColgan T., Speiser A., Macke J. H., Turaga S. C., Mineault P., Rupprecht P., , “Community-based benchmarking improves spike rate inference from two-photon calcium imaging data,” PLoS computational biology, vol. 14, no. 5, p. e1006157, 2018. 10.1371/journal.pcbi.1006157 PMC5997358 29782491

[116] Magland J., Jun J. J., Lovero E., Morley A. J., Hurwitz C. L., Buccino A. P., Garcia S., and Barnett A. H., “Spikeforest, reproducible web-facing ground-truth validation of automated neural spike sorters,” Elife, vol. 9, p. e55167, 2020. 10.7554/eLife.55167 PMC7237210 32427564

[117] Blankertz B., Muller K.-R., Curio G., Vaughan T. M., Schalk G., Wolpaw J. R., Schlogl A., Neuper C., Pfurtscheller G., Hinterberger T., , “The bci competition 2003: progress and perspectives in detection and discrimination of eeg single trials,” IEEE transactions on biomedical engineering, vol. 51, no. 6, pp. 1044–1051, 2004. 10.1109/TBME.2004.826692 15188876

[118] Wei X., Faisal A. A., Grosse-Wentrup M., Gramfort A., Chevallier S., Jayaram V., Jeunet C., Bakas S., Ludwig S., Barmpas K., , “2021 beetl competition: Advancing transfer learning for subject independence and heterogenous eeg data sets,” in NeurIPS 2021 Competitions and Demonstrations Track, pp. 205–219, PMLR, 2022.

[119] Turishcheva P., Fahey P. G., Vystrčilová M., Hansel L., Froebe R., Ponder K., Qiu Y., Willeke K. F., Bashiri M., Baikulov R., Zhu Y., Ma L., Yu S., Huang T., Li B. M., De Wulf W., Kudryashova N., Hennig M. H., Rochefort N. L., Onken A., Wang E., Ding Z., Tolias A. S., Sinz F. H., and Ecker A. S., “Retrospective for the dynamic sensorium competition for predicting large-scale mouse primary visual cortex activity from videos,” in Advances in Neural Information Processing Systems, vol. 37, 2024.

[120] Radford A., Wu J., Child R., Luan D., Amodei D., Sutskever I., , “Language models are unsupervised multitask learners,” OpenAI blog, vol. 1, no. 8, p. 9, 2019.

[121] Achiam J., Adler S., Agarwal S., Ahmad L., Akkaya I., Aleman F. L., Almeida D., Altenschmidt J., Altman S., Anadkat S., , “Gpt-4 technical report,” arXiv *preprint arXiv:2303.08774*, 2023.

[122] Kaplan J., McCandlish S., Henighan T., Brown T. B., Chess B., Child R., Gray S., Radford A., Wu J., and Amodei D., “Scaling laws for neural language models,” arXiv *preprint arXiv:2001.08361*, 2020.

[123] Cherti M., Beaumont R., Wightman R., Wortsman M., Ilharco G., Gordon C., Schuhmann C., Schmidt L., and Jitsev J., “Reproducible scaling laws for contrastive language-image learning,” in Proceedings of the IEEE/CVF conference on computer vision and pattern recognition, pp. 2818–2829, 2023.

[124] Antonello R., Vaidya A., and Huth A., “Scaling laws for language encoding models in fmri,” Advances in Neural Information Processing Systems, vol. 36, pp. 21895–21907, 2023. PMC11258918 39035676

[125] Sato M., Tomeoka K., Horiguchi I., Arulkumaran K., Kanai R., and Sasai S., “Scaling law in neural data: Non-invasive speech decoding with 175 hours of eeg data,” arXiv *preprint arXiv:2407.07595*, 2024.

[126] Banville H., Benchetrit Y., d’Ascoli S., Rapin J., and King J.-R., “Scaling laws for decoding images from brain activity,” arXiv *preprint arXiv:2501.15322*, 2025.

[127] Jiang L. P., Chen S., Tanumihardja E., Han X., Shi W., Shea-Brown E., and Rao R. P., “Data heterogeneity limits the scaling effect of pretraining neural data transformers,” bioRxiv, pp. 2025–05, 2025.

[128] Safaie M., Chang J. C., Park J., Miller L. E., Dudman J. T., Perich M. G., and Gallego J. A., “Preserved neural dynamics across animals performing similar behaviour,” Nature, vol. 623, no. 7988, pp. 765–771, 2023. 10.1038/s41586-023-06714-0 PMC10665198 37938772

